# Supplementary figures and images for: Polymorphism in a Neotropical toad species: ontogenetic, populational and geographic approaches to chromatic variation in Proceratophrys cristiceps (Müller, 1883) (Amphibia, Anura, Odontophrynidae)
Source: PeerJ. 2022 Mar 25;10:e12879. doi: 10.7717/peerj.12879 (PMC8958967; doi:10.7717/peerj.12879)

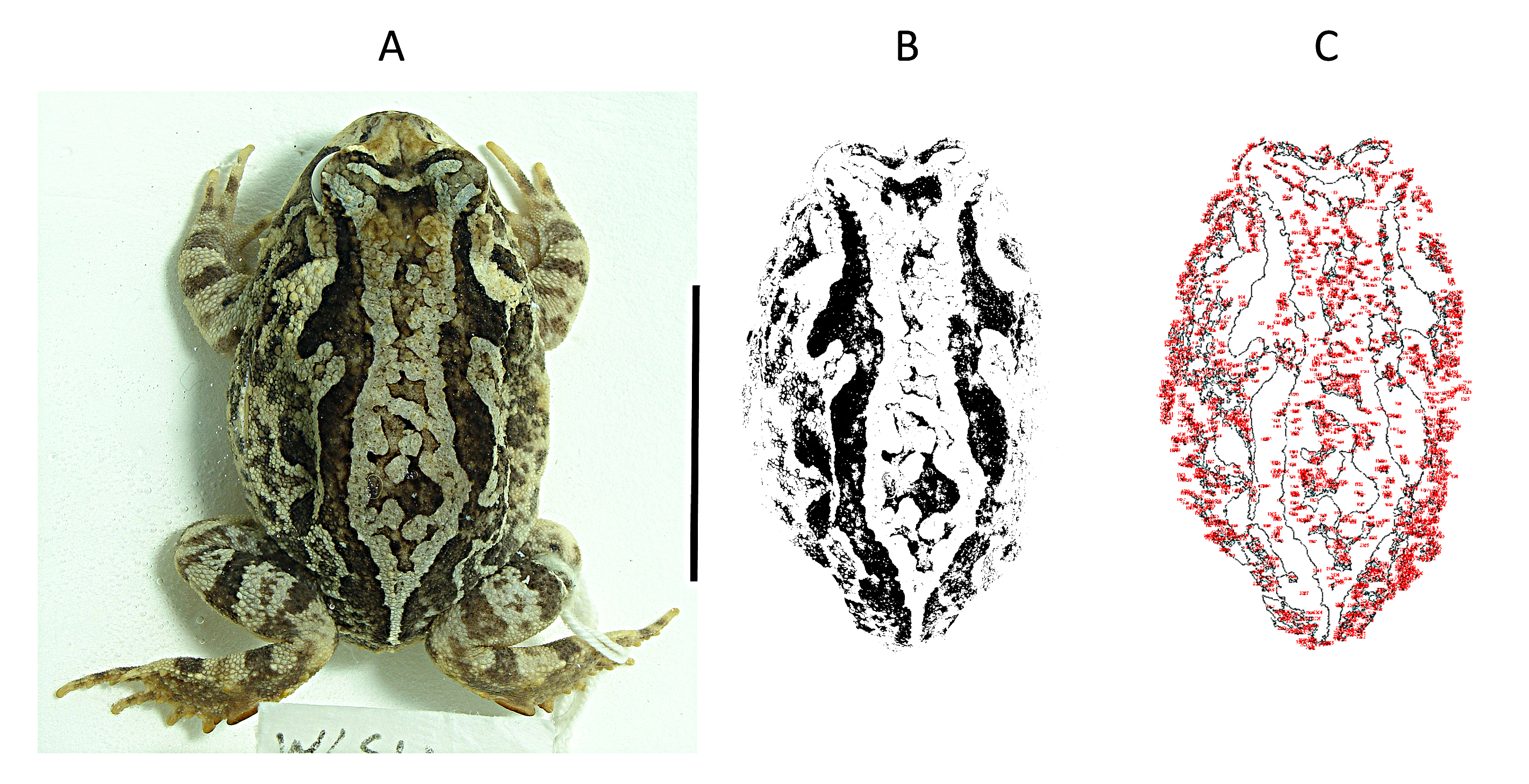

Supplement: Supplemental Information 1 — (B) Characteristic dorsal (8-bit) chromatic pattern. (C) Total area of spots (red colour) calculated along the dorsal surface of the specimen. Measurements sets: area; minimum and maximum grey value; mean grey value. Bar: 56 mm. Photo credit: Kleber Vieira. [file peerj-10-12879-s001.png]

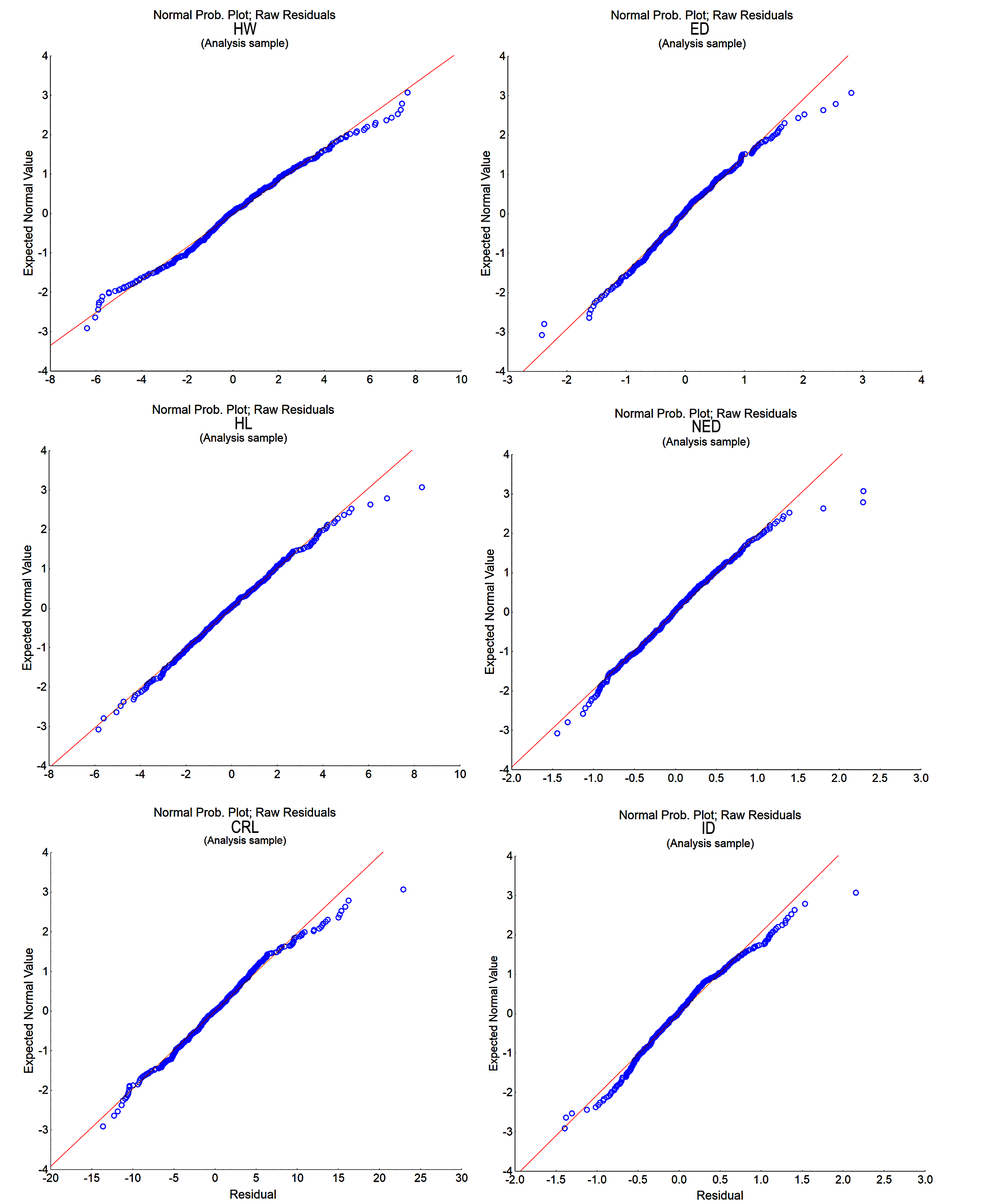

Supplement: Supplemental Information 2 [file peerj-10-12879-s002.png]

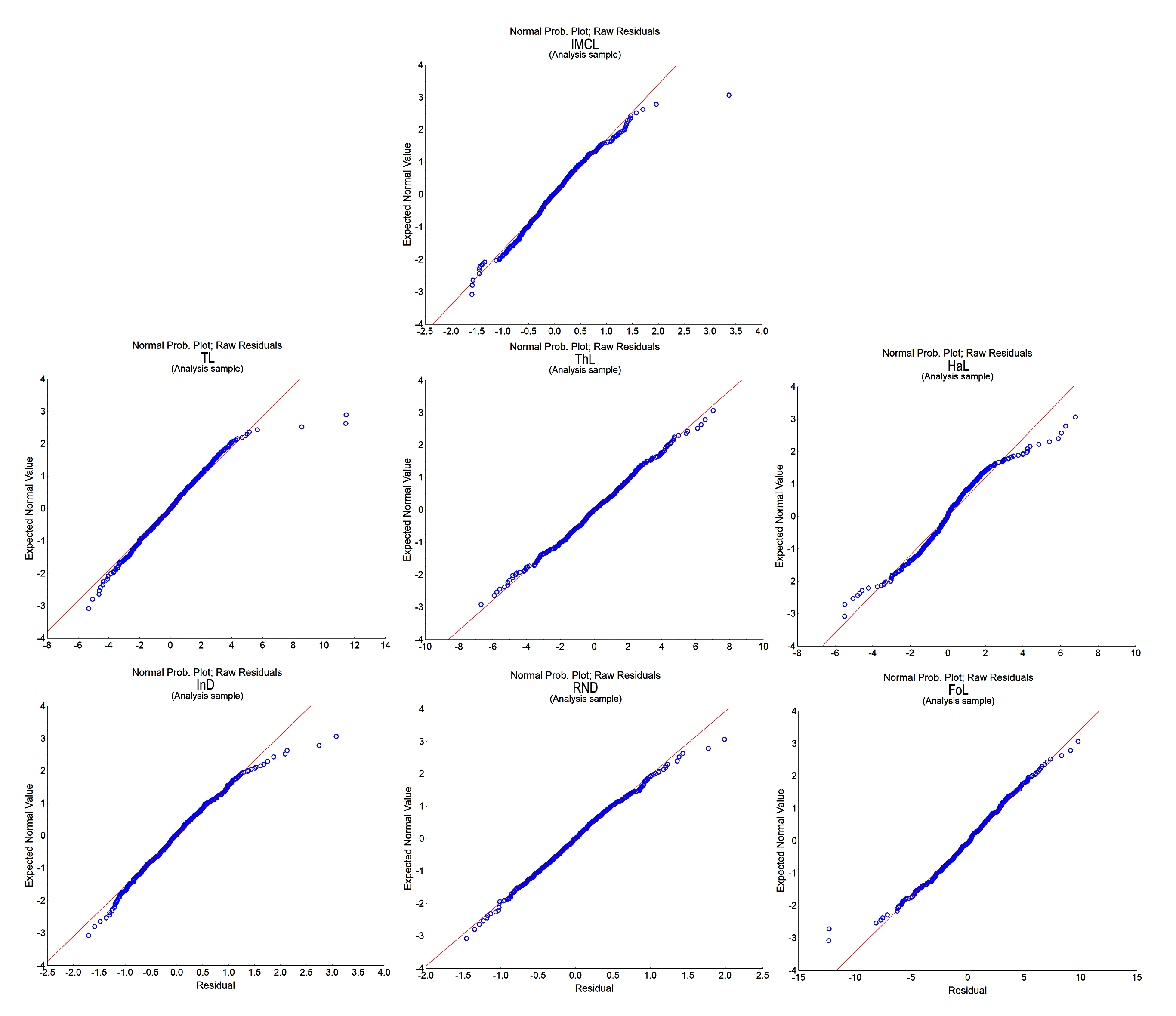

Supplement: Supplemental Information 3 [file peerj-10-12879-s003.png]

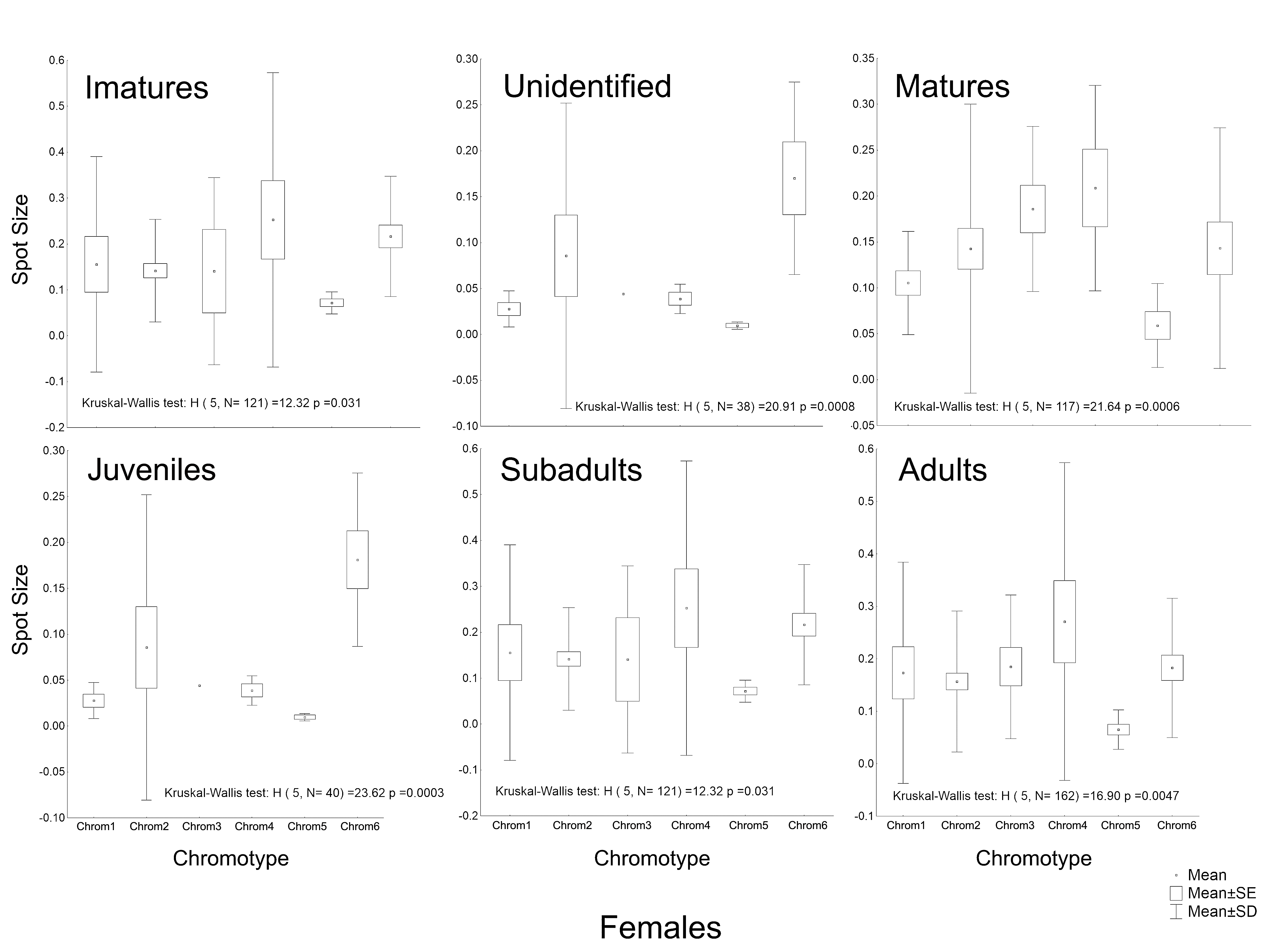

Supplement: Supplemental Information 4 — Chrom5 individuals are significantly different (α = 0.05) from the other chromotypes, demonstrating smaller spots. Curiously, females generally demonstrated a greater average spot size compared to males. [file peerj-10-12879-s004.png]

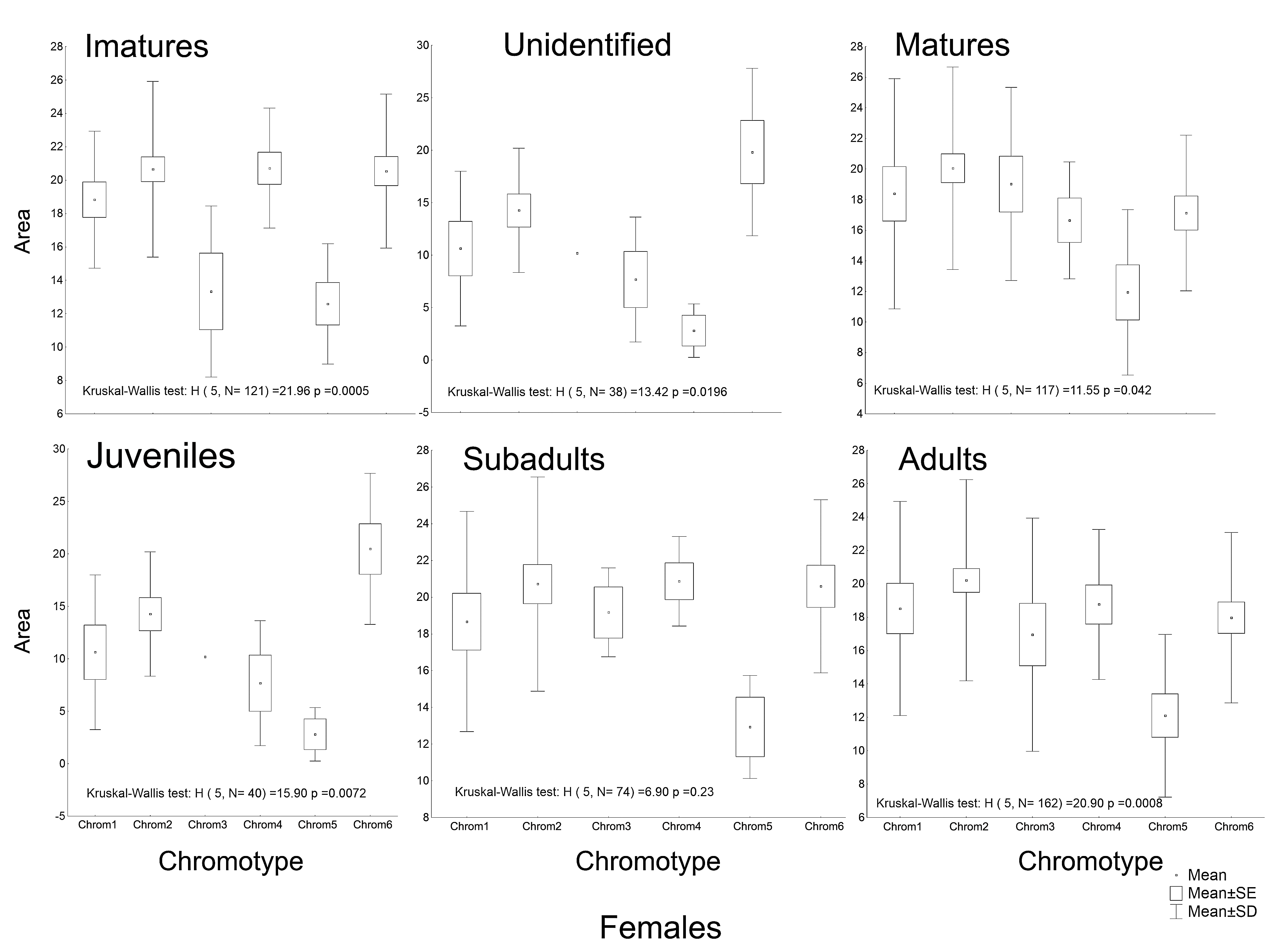

Supplement: Supplemental Information 5 — Chrom5 individuals are significantly different (α = 0.05) from the other chromotypes, demonstrating smaller spots that are located farther apart from one another. [file peerj-10-12879-s005.png]

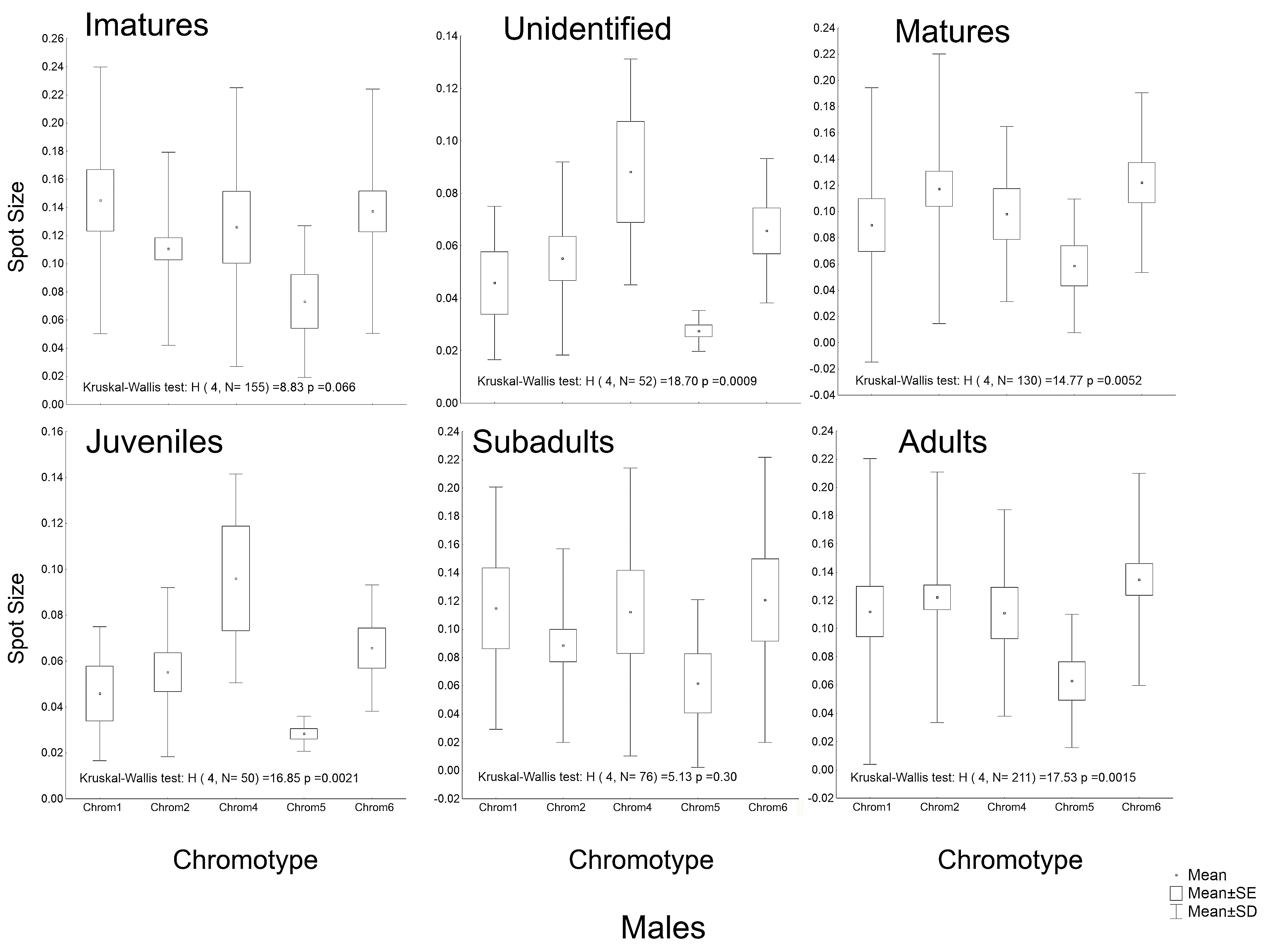

Supplement: Supplemental Information 6 — Chrom 5 individuals are significantly different (α = 0.05) from the other chromotypes, demonstrating smaller spots. Some values not observed. [file peerj-10-12879-s006.png]

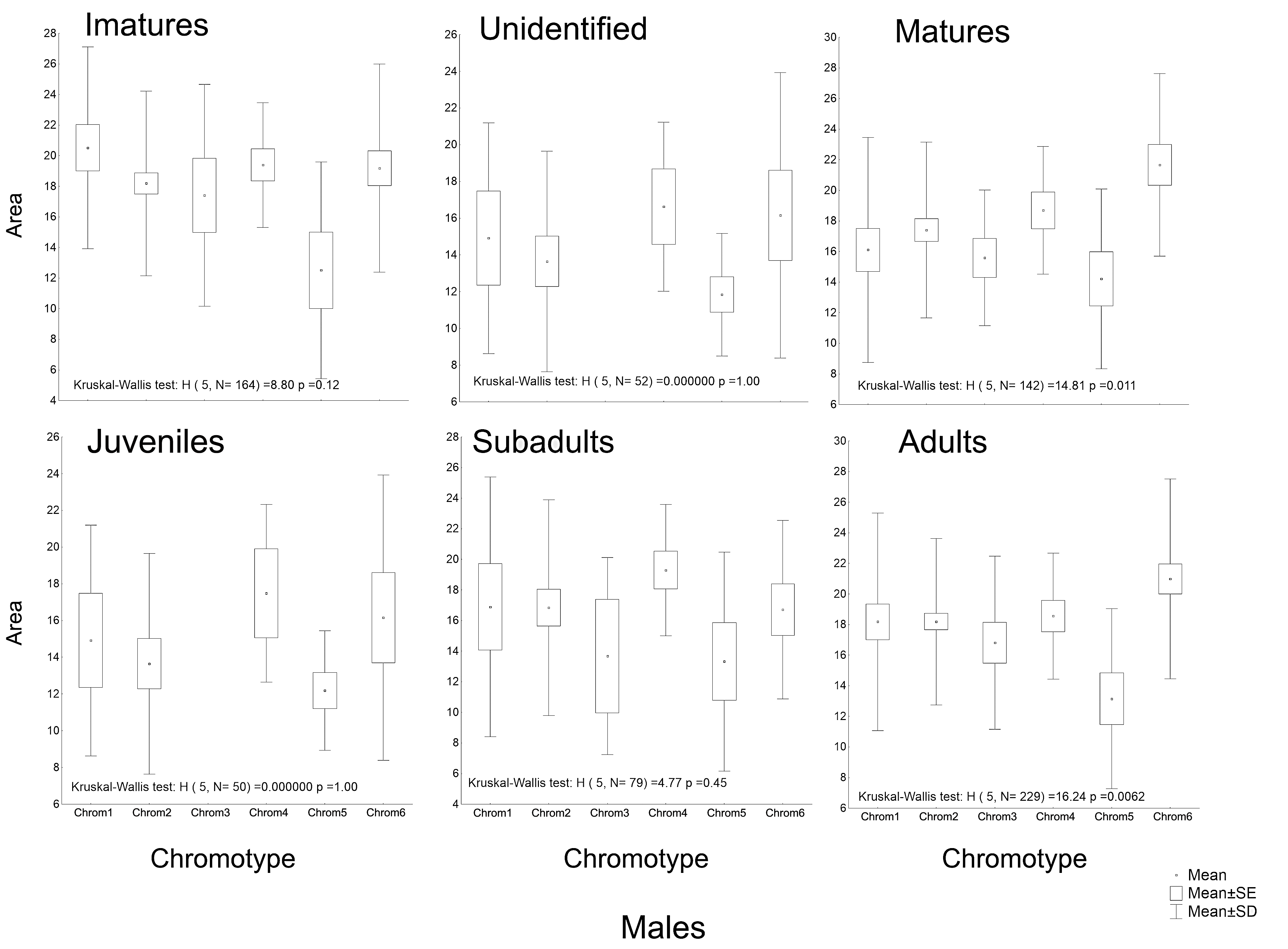

Supplement: Supplemental Information 7 — Chrom5 individuals are significantly different (α = 0.05) from the other chromotypes, demonstrating smaller spots that are located farther apart from one another. Males exhibit a smaller average distribution area as compared to females. Some values not observed. [file peerj-10-12879-s007.png]

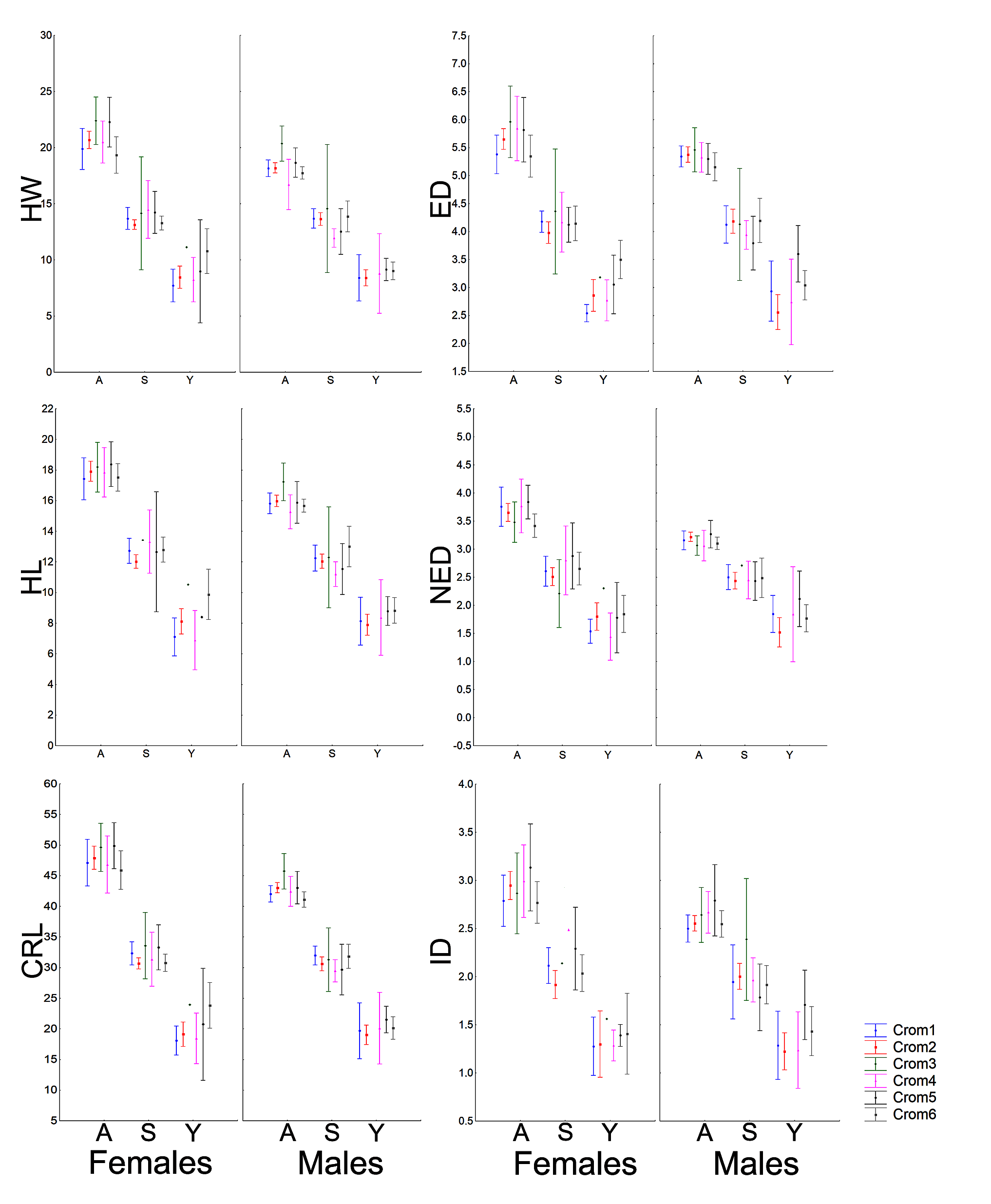

Supplement: Supplemental Information 8 — indicating that males and females are equivalent when comparing them in terms of ontogenetic classes (post-larval). Wilks’ lambda = 0.81; F(117, 4400, 6) = 1.05; p = 0.34. Vertical bars denote 0.95 confidence intervals (weighted marginal means, some means not observed). [file peerj-10-12879-s008.png]

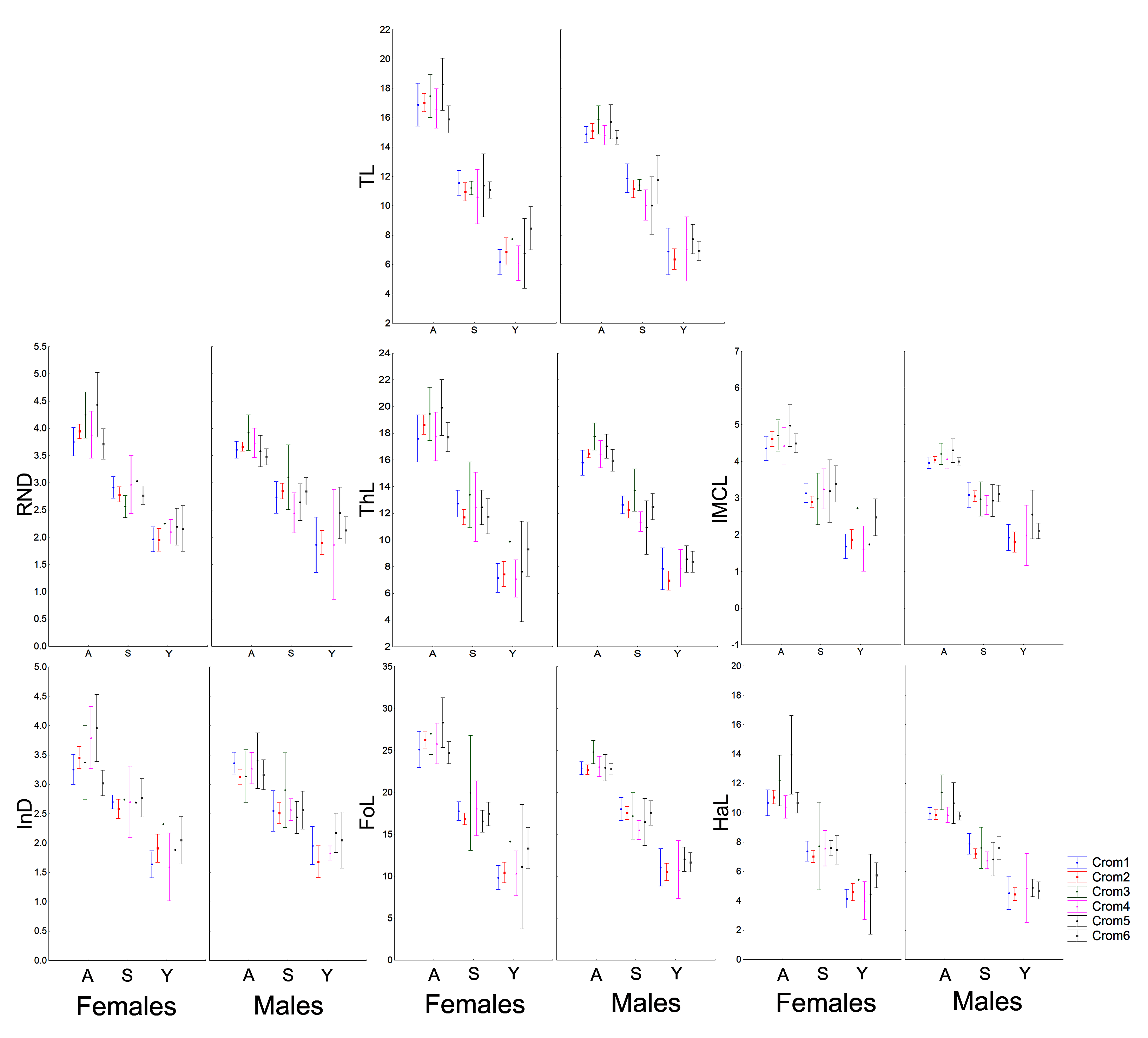

Supplement: Supplemental Information 9 — indicating that males and females were equivalent when comparing ontogenetic classes (post-larval). Wilks’ lambda = 0.81; F(117, 4400, 6) = 1.05; p = 0.34. Vertical bars denote 0.95 confidence intervals (weighted marginal means, some means not observed). [file peerj-10-12879-s009.png]

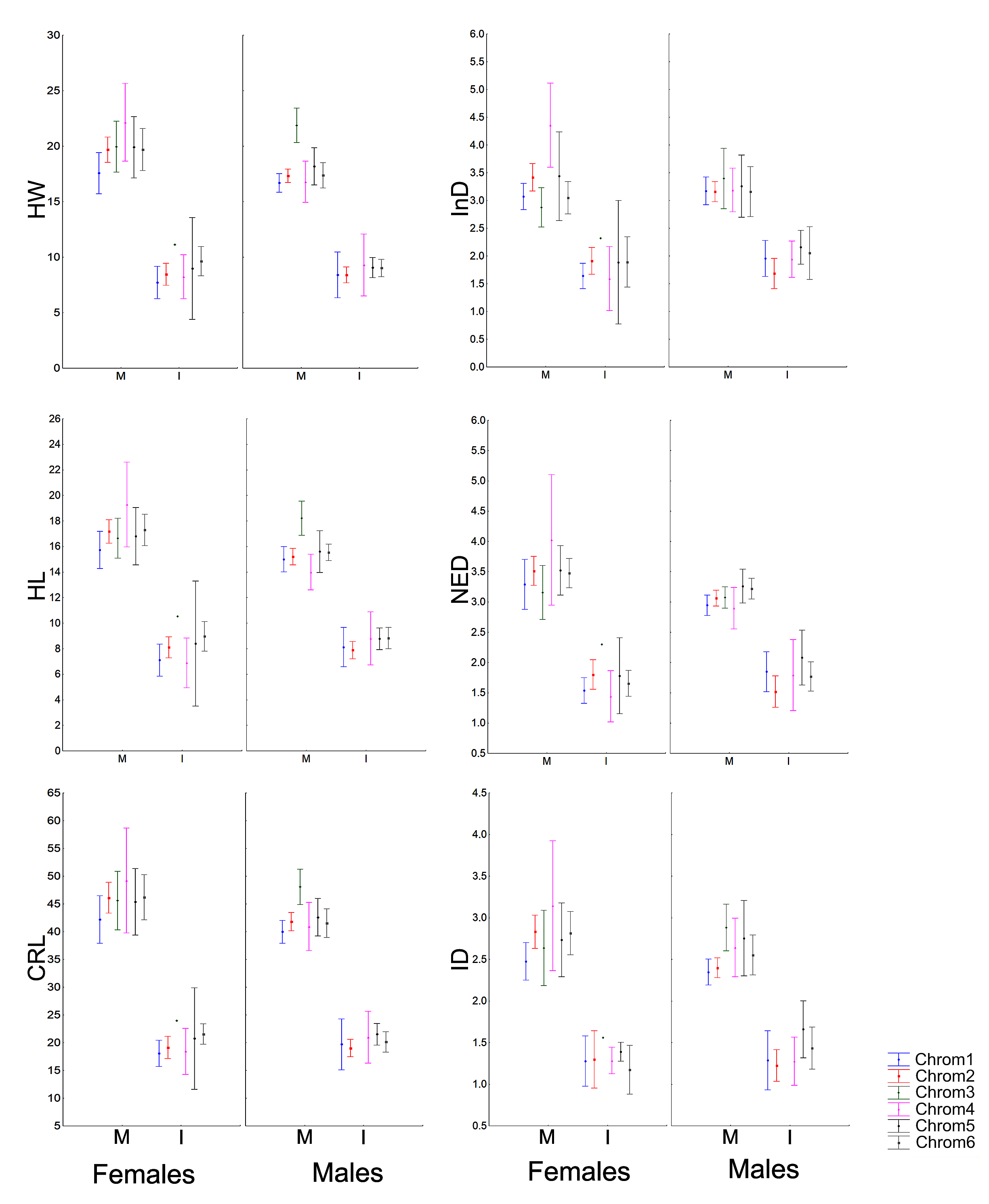

Supplement: Supplemental Information 10 — indicating that the males and females were equivalent when comparing maturity classes (Immature and Mature). Wilks’ lambda = 0.80; F(52, 1218, 2) = 1.33; p = 0.063. Vertical bars denote 0.95 confidence intervals (weighted marginal means, some means not observed). [file peerj-10-12879-s010.png]

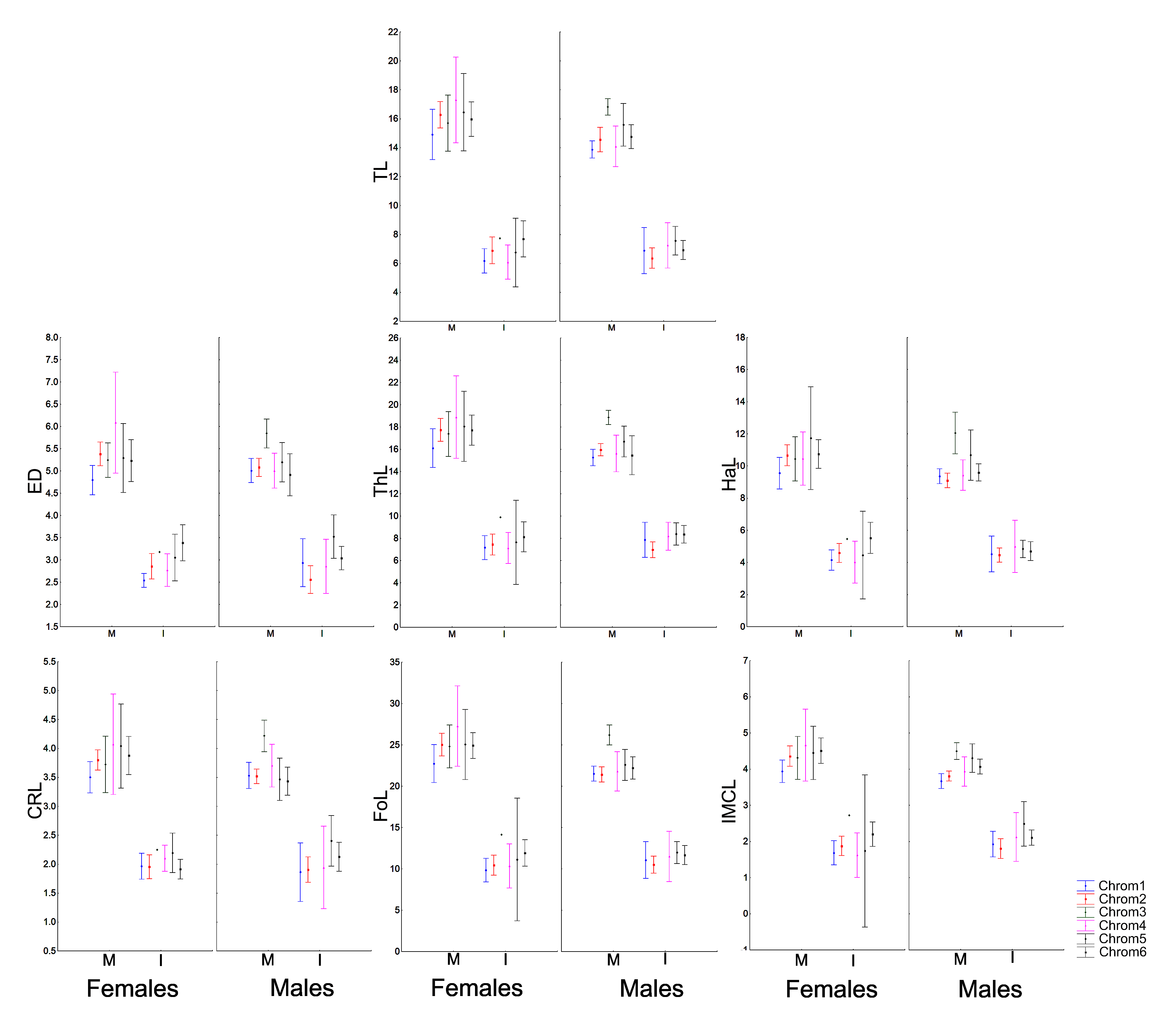

Supplement: Supplemental Information 11 — indicating that the males and females were equivalent when comparing maturity classes (Immature and Mature). Wilks’ lambda = 0.80; F(52, 1218, 2) = 1.33; p = 0.063. Vertical bars denote 0.95 confidence intervals (weighted marginal means, some means not observed). [file peerj-10-12879-s011.png]

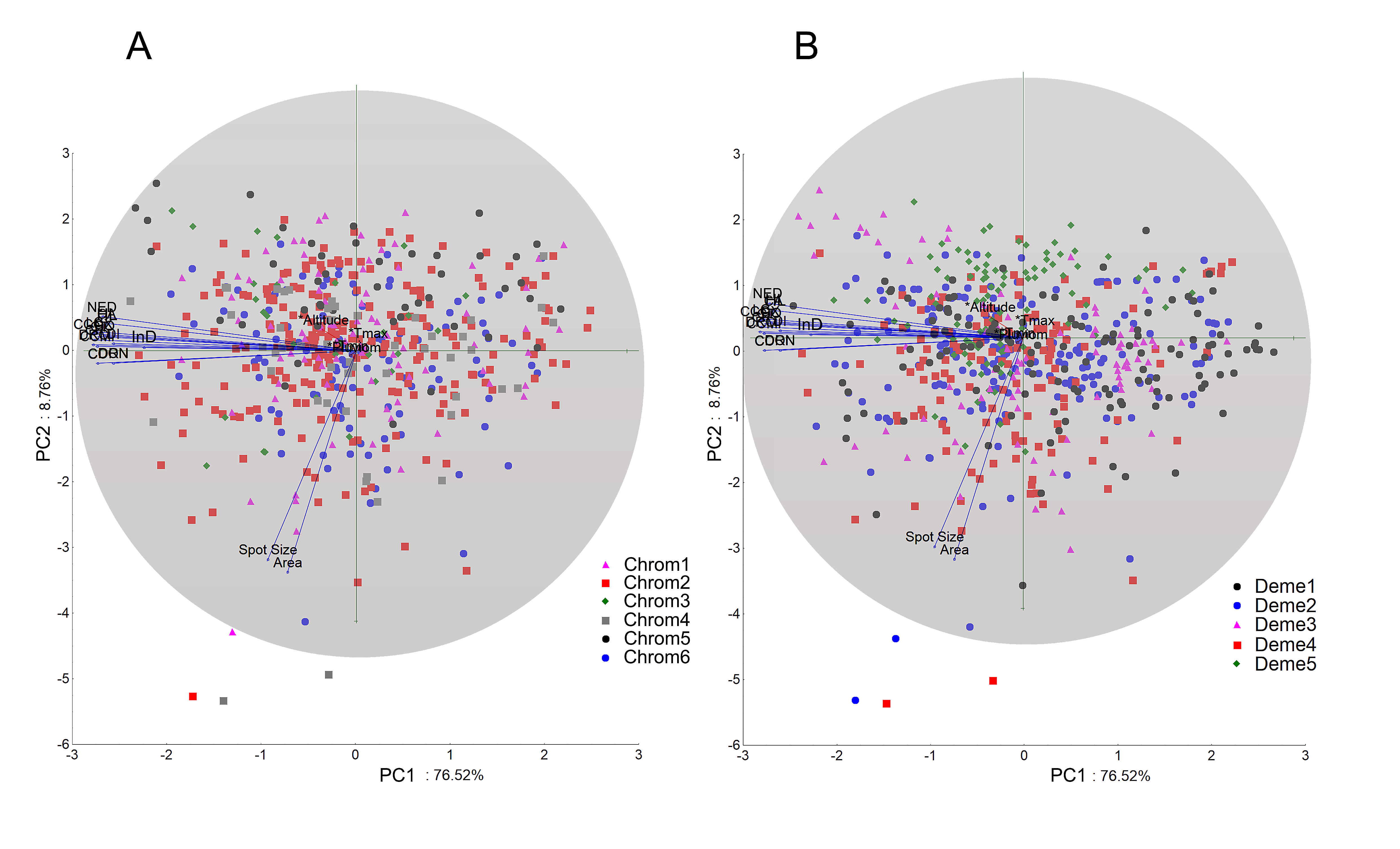

Supplement: Supplemental Information 12 — PC1 is correlated with size dimensions, whereas PC2 is correlated with saturation. It is possible to verify that Chrom5 and Dem5 are more concentrated and distributed along the superior portion of the second component, suggesting the presence of low saturated specimens. The environmental predictors did not explain the chromatic variance observed, indicating the existence of underlying operating factors. [file peerj-10-12879-s012.png]

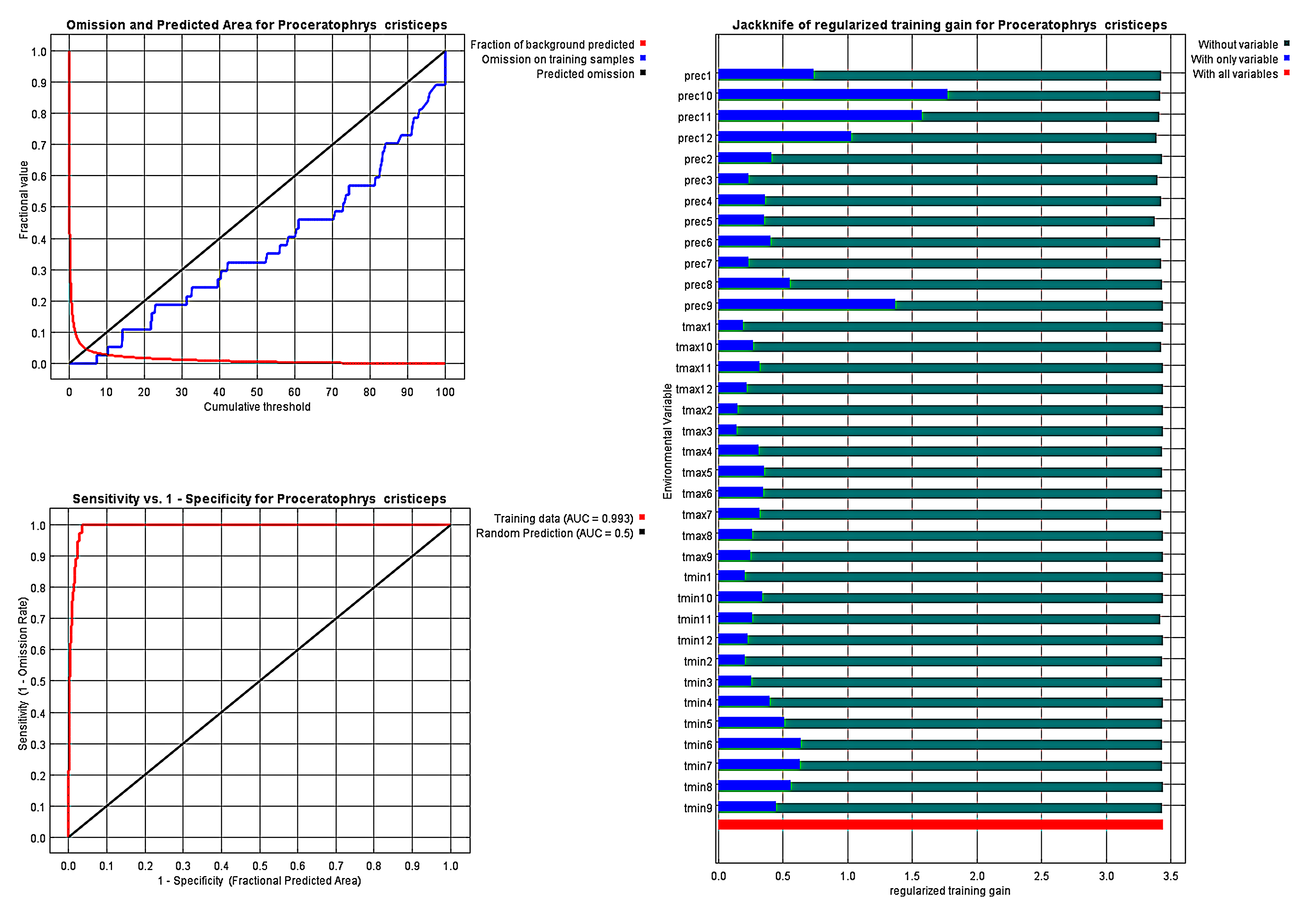

Supplement: Supplemental Information 13 — The data indicated that the species is typical of the Caatinga, being found with greater probability in the tropical savanna and semi-arid climate zones of this biome, according to the Köppen–Geiger classification. [file peerj-10-12879-s013.png]

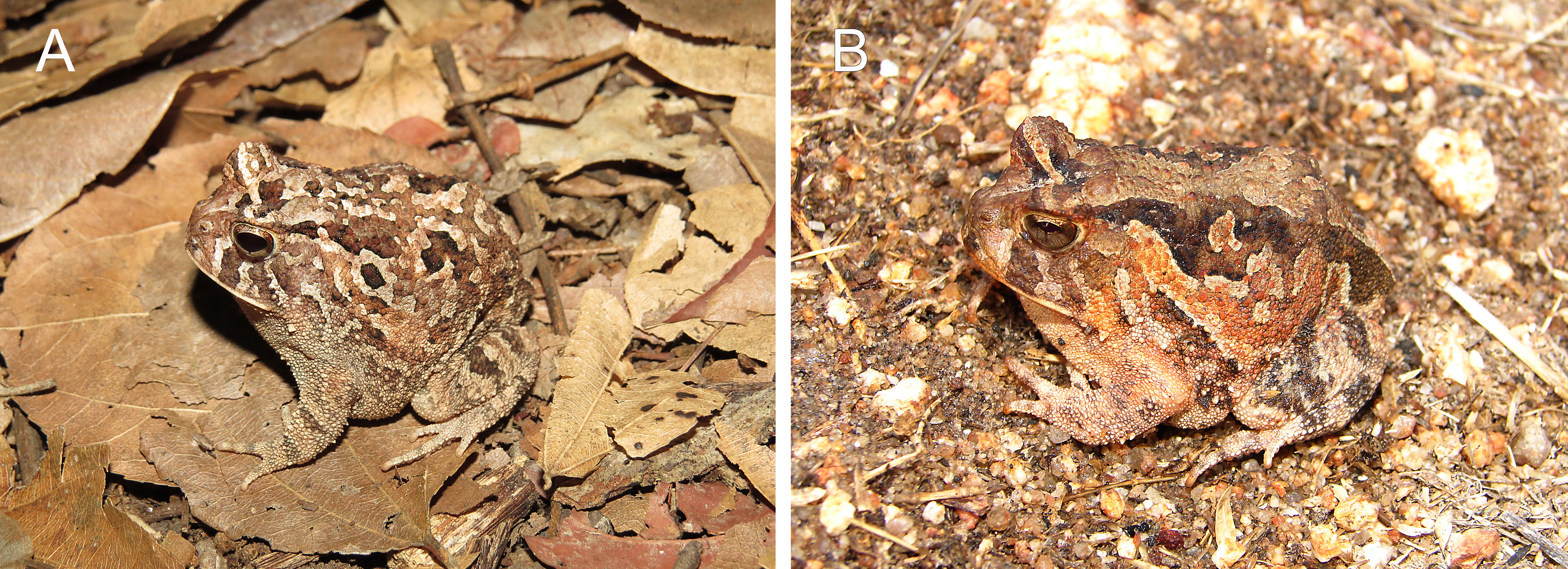

Supplement: Supplemental Information 14 — (A) Chrom1 and (B) Chrom2. The contrast of the animals’ coloring in relation to the soil suggests adaptive reinforcement of the individual survival capacity (crypsis). Photo credit: Washington L. S. Vieira. [file peerj-10-12879-s014.png]

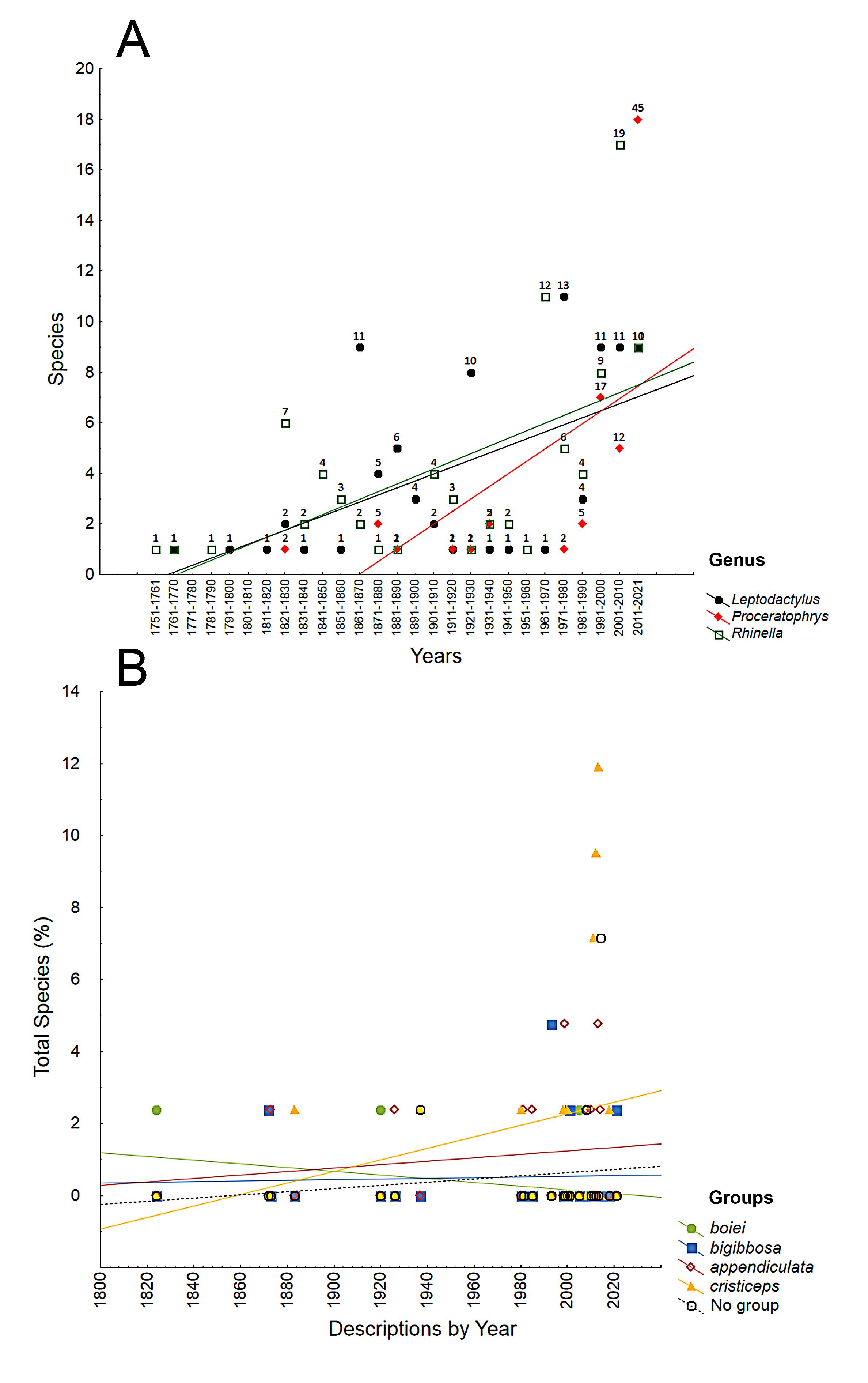

Supplement: Supplemental Information 15 — The lines represent least squares regressions, while the numbers over the dots represent the periodic rate (%) of the descriptions (A). We found that the species of the genera Leptodactylus and Rhinella increased at similar rates over the decades, being later surpassed by Proceratophrys due to its faster rate of annual descriptions (A). When compared among congeneric groups (B), the highest description rates are observed in the cristiceps group. The bigibbosa group has been reasonably stable, but the boiei group rate has declined in relation to the total. Data obtained from Frost, D. R. (2021). Amphibian Species of the World: an Online Reference. Version 6.1. [file peerj-10-12879-s015.png]

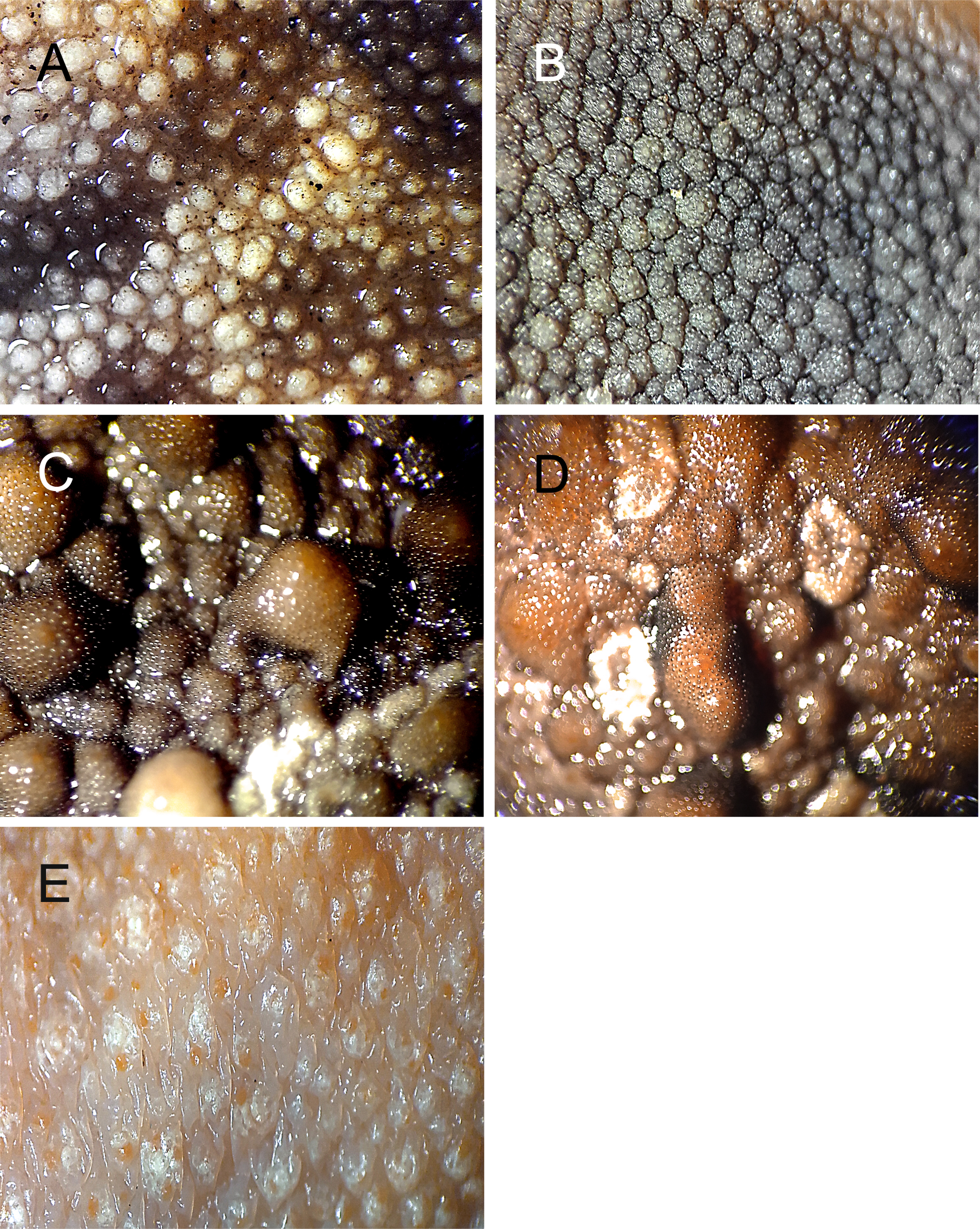

Supplement: Supplemental Information 16 — Gular region: slightly globular and smooth (A) or rough (B); dorsal glandular nodules varying in shape and size (C and D); ventral posterior portion: elongated and flattened (E). Photo credit: Kleber Vieira. [file peerj-10-12879-s016.png]

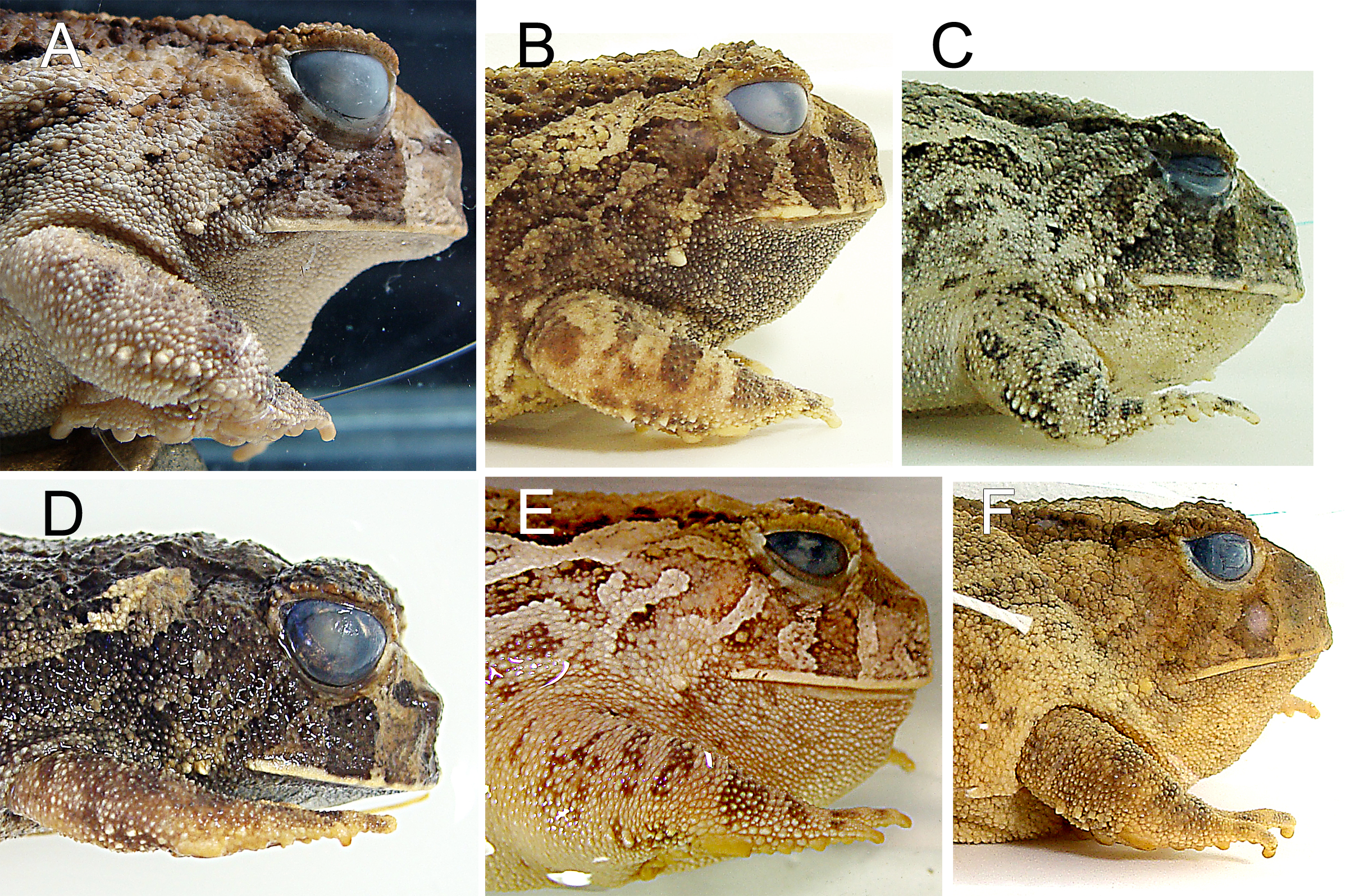

Supplement: Supplemental Information 17 — on the outer portion of the right forearm and buccal (and/or subocular) commissure in specimens of P. cristiceps. A (WLSV 1474); B (WLSV 4095); C (WLSV 4791); D (UFPB 23174); E (UFPB 7214) e F (KSV 237). Photo credit: Kleber Vieira. [file peerj-10-12879-s017.png]
